# Biofilm Prevention and Removal in Non-Target *Pseudomonas* Strain by *Siphovirus*-like Coliphage

**DOI:** 10.3390/biomedicines12102291

**Published:** 2024-10-09

**Authors:** Leonardo Martín Pérez, Olesia Havryliuk, Nury Infante, Maite Muniesa, Jordi Morató, Ruslan Mariychuk, Tzanko Tzanov

**Affiliations:** 1 Laboratory of Sanitary and Environmental Microbiology (MSMLab)-UNESCO Chair on Sustainability, Universitat Politècnica de Catalunya-BarcelonaTech, R/Sant Nebridi, 22, GAIA Building (TR14), 08222 Terrassa, Spain; leonardo.martin.perez@upc.edu (L.M.P.); o.havryliuk@imv.org.ua (O.H.); nury.gineth.infante@upc.edu (N.I.); jordi.morato@upc.edu (J.M.); 2 Grup de Biotecnologia Molecular i Industrial, Departament d’Enginyeria Química, Universitat Politècnica de Catalunya (UPC-BarcelonaTech), Rambla de Sant Nebridi 22, 08222 Terrassa, Spain; tzanko.tzanov@upc.edu; 3 Department of Extremophilic Microorganisms Biology, Zabolotny Institute of Microbiology and Virology, National Academy of Sciences of Ukraine, 03143 Kyiv, Ukraine; 4 Departament de Genètica, Microbiologia i Estadística, Universitat de Barcelona, Diagonal 643 (Annex. Floor 0), 08028 Barcelona, Spain; mmuniesa@ub.edu; 5 Department of Ecology, Faculty of Humanities and Natural Sciences, University of Presov, 08001 Presov, Slovakia

**Keywords:** antimicrobial-resistant bacteria, somatic coliphage, biofilm inhibition and eradication, *Siphovirus*-like phages

## Abstract

**Background/Objectives.** Bacteriophages have gained significant interest as a potential solution to combat harmful bacteria, especially in the fight against antimicrobial resistance. With the rise in drug-resistant microorganisms, the medical community is increasingly exploring new alternatives to traditional antibiotics, and bacteriophages offer several advantages in this regard. However, phage applications still face some challenges, such as host specificity. **Methods.** In this study, a somatic *Siphovirus*-like coliphage (SOM7) was tested for inhibiting the biofilm-forming capacity of the non-target strain *Pseudomonas aeruginosa* (ATTC 10145). The phage-sensitive strain *E. coli* WG5 was used as a control. The selected microorganisms were first tested for growth in the presence of SOM7 at three different concentrations (10^5^, 10^7^, and 10^9^ PFU/mL). **Results.** As expected, the phage-sensitive *E. coli* WG5 was fully inhibited by the coliphage, and no phage-related affection on the growth rate was observed for the SOM7-resistant *P. aeruginosa*. More notably, increasing concentrations of SOM7 significantly reduced both the biofilm-forming capacity and the amount of pre-established bacterial biofilm of the phage-insensitive *P. aeruginosa* (24.9% and 38.8% reduction in the biofilm-forming ability, and 18.8% and 28.0% biofilm degradation for 10^7^ PFU/mL and 10^9^ PFU/mL SOM7, respectively; *p* < 0.05). These results were supported by transmission electron microscopy (TEM) imaging, providing unprecedent evidence for the interaction of the somatic coliphage with the non-host strain. **Conclusions.** Although more studies in other biofilm models are necessary, our results show for the very first time that bacteriophages could potentially be used as an alternative to achieve desired anti-biofilm and biofilm-degrading activity in non-host bacterial strains.

## 1. Introduction

The rapid emergence and spread of antimicrobial-resistant bacteria represents a worldwide crisis, especially in low- and middle-income countries wherein harmful pathogens cause significant mortality and morbidity [1,2,3]. Resistance development is facilitated by the biofilm-forming ability of different bacterial strains, which protects the microorganisms by enclosing them in a complex extracellular matrix. Biofilm formation is the key determinant in medical device-associated infections, e.g., catheters, stents, implants, prostheses, and medical monitoring devices [4,5]. Moreover, bacteria living in biofilms are also more resistant to antibiotics and different physicochemical treatments, even those highly aggressive [6,7]. Therefore, the establishment of biofilms represents an undesirable and unwelcome phenomenon since bacterial attachment to biotic or abiotic surfaces leads to the formation of an intricate microbial community encompassing not only clinical but also environmental concerns due to the spread of antimicrobial genes [2,3].

The growth of bacteria living in biofilms attached to both synthetic and natural materials is mainly limited to the availability of surrounding nutrients [4]. In addition, different studies have demonstrated that the ability of bacteria to develop biofilms depends on several factors, including the presence of xenobiotic agents, quorum sensing (QS) signals, and environmental stressors that trigger biofilm-developing mechanisms. Even the morphology of the cells plays an important role in the capacity of different microorganisms to develop biofilms [8,9].

Among microorganisms, Gram-negative bacteria show great potential in forming biofilms, and this ability is also associated with an increased microbial pathogenicity. *Escherichia coli* and *P. aeruginosa* are two of the most frequent biofilm-forming pathogens associated with an increasing number of nosocomial infections and clinical infectious diseases, as well as different environmental manifestations [10,11]. Additionally, both bacteria usually refer to broad antibiotic resistance profiles. Therefore, studies evaluating new strategies to counteract the growth and establishment of microbial biofilms involving *E. coli* and *P. aeruginosa* species are of utmost importance.

During the last few decades, bacteriophages have gained a lot of interest as potential alternatives to antibiotics for the control of harmful bacteria. In addition, these bacteria-infecting viruses have also been explored as anti-biofilm agents, as they are capable of lysing host bacteria in microbial communities (i.e., biofilms) [4]. However, phage applications still face some challenges, such as host specificity and the emergence of bacterial resistance to phage infection. Moreover, bacteria have evolved, acquiring several mechanisms to interfere with a bacteriophage’s infection, such as phage adsorption inhibition, the prevention of phage entry, and phage exclusion [12]. Interestingly, phages have also developed some counterstrategies to circumvent bacterial anti-phage mechanisms, such as the ability to attach to new receptors or binding sites on the bacterial cell surface and digging for specific receptors [13]. In such a sense, most of the information known about phage–bacteria interaction comes from studies involving the classical approach, i.e., testing phage infection in phage-sensitive bacteria. But there is no information about potential interactions and the associated effects of bacteriophages contacting a non-host strain. Therefore, this work is pioneering in exploring this behaviour and how even faint or unspecific interaction can disturb biofilm establishment of phage-insensitive strains. Hence, understanding the complex dynamics of host but also non-host bacteria–phage interaction is a preliminary step towards the development of new strategies to defeat multidrug-resistant bacterial infections, among other unexplored phage-based biotechnological applications [14,15,16,17,18].

## 2. Materials and Methods

### 2.1. Bacteriophage

The somatic coliphage SOM7 isolated from seawater, as previously described [19], was investigated in this study. According to its morphological characteristics, this phage has been classified as *Siphovirus*-like according to the updated taxonomy [20]. The phage suspension was obtained and purified following the procedure for somatic coliphage propagation described in the ISO 10507-2 standard and quantified by the double-layer agar technique [21]. The pure phage solution was obtained via ultracentrifugation (30,000 rpm) at 4 °C [22]. The supernatant (containing bacterial proteins) was carefully removed. The phage pellet was then resuspended in PBS buffer. The final bacteriophage suspension contained 10^10^ plaque-forming units per millilitre (PFU/mL) and was stored at 4 °C until use.

### 2.2. Bacterial Strains and Growth Conditions

*P. aeruginosa* ATCC 10145 (accession number VAOQ00000000.1) was purchased from the American Type Culture Collection (ATCC^®^ LGC Standards, Barcelona, Spain). *E. coli* WG5, the reference host recommended by the International Organisation for Standardisation (ISO) to detect somatic coliphages, was used here as the control strain for SOM7 infection [23]. Bacterial stock cultures (500 µL culture, 500 µL glycerol) were stored at −70 °C in sterile Eppendorf^®^ tubes (Hamburg, Germany). The cells were grown overnight (16–18 h) in Luria–Bertani (LB) broth (Sigma-Aldrich, St. Louis, MO, USA) at 35 °C and 230 rpm agitation. Fresh overnight cultures were used as the inoculum for further tests.

### 2.3. Antimicrobial Activity

The effect of the SOM7 coliphage on the growth of the tested bacteria was determined by measuring the optical density recorded at 600 nm (OD_600_) over 18 h at 35 ± 1 °C using a TECAN Infinite M200 Microplate Reader (GENios-Tecan, Männedorf, Switzerland) [24]. The growth curves were performed in LB broth in the absence (control) or presence of the coliphage at three different concentrations (10^5^, 10^7^, and 10^9^ PFU/mL). Fresh overnight (16–18 h) cultures of *E. coli* WG5 and *P. aeruginosa* (ATCC 10145) were adjusted to an OD_600_ ≈ 0.6 in sterile saline solution (NaCl 0.85% *w*/*v*) and used as the test inoculum (10 μL) in a 96-well microtitre plate (Sarstedt, Nümbrecht, Germany) with a final volume of 250 μL. The curves were obtained in triplicate (*n* = 3).

### 2.4. Anti-Biofilm Assay

The biofilm inhibition assay was carried out using the classical spectrophotometric approach based on crystal violet staining [25]. Bacterial strains (*E. coli* WG5 and *P. aeruginosa* ATCC 10145) were grown in LB broth in the absence (control) or presence of the somatic coliphage at three different concentrations (10^5^, 10^7^, and 10^9^ PFU/mL) in a sterile 96-well microtitre plate (Sarstedt, Nümbrecht, Germany). Fresh overnight cultures of the tested bacteria grown in LB broth and adjusted to an OD_600_ ≈ 0.6 in sterile saline solution (NaCl 0.85% *w*/*v*) were used as the test inoculum (10 µL). The plate was incubated for 24 h at 35 ± 1 °C in a culture chamber without agitation. After incubation, the medium was removed along with the planktonic bacteria, and the attached cells were washed four times with sterile distilled water. Further, the plate was inverted, tapped gently to remove the excess of liquid, and dried for 30 min at 35 ± 1 °C in a culture chamber. Afterwards, 250 µL of a 0.1% (*w*/*v*) crystal violet (Sigma-Aldrich, St. Louis, MO, USA) staining solution prepared in MilliQ water was added to each well, and the plate was incubated at room temperature for 15 min. After staining, the stained cells in the wells were washed four times with sterile distilled water, and the plate was inverted and placed on a paper towel inside a culture chamber at 35 ± 1 °C for drying during another 30 min. Further, crystal violet extraction was performed by adding 250 µL of 30% (*v*/*v*) acetic acid to each well and leaving the plate to incubate for 15 min at room temperature. Finally, 200 µL of the clear solution was transferred to a new 96-well microtitre plate, and the absorbance was measured at 550 nm in a TECAN Infinite M200 Microplate Reader (GENios-Tecan, Männedorf, Switzerland). The assay was repeated three times in four replicates.

### 2.5. Biofilm-Degrading Assay

The biofilm degradation ability of the SOM7 coliphage against *E. coli* WG5 and *P. aeruginosa* (ATCC 10145) strains was evaluated by staining the remaining biofilm after phage treatments with crystal violet [26]. Briefly, the tested bacteria were grown in LB broth (without the presence of the phage) in a 96-well microtitre plate for 48 h at 35 ± 1 °C in a culture chamber and without agitation to allow for biofilm establishment. After incubation, the planktonic cells were carefully removed before being replaced with 250 μL of a coliphage solution containing 10^5^, 10^7^, or 10^9^ PFU/mL prepared in sterile LB broth. In addition, sterile LB broth was used as a control. Later, the microplate was incubated in a culture chamber at 35 ± 1 °C for another 24 h. After that, the medium was carefully removed, and the remained attached cells were washed four times with sterile distilled water. Further, the plate was inverted, tapped gently to remove excess liquid, and dried for 30 min at 35 ± 1 °C in a culture chamber. Finally, 250 µL of a 0.1% (*w*/*v*) crystal violet (Sigma-Aldrich, St. Louis, MO, USA) staining solution was used to reveal the biofilms, as previously described. Two hundred (200 µL) aliquots of the clear violet solution were transferred to a new 96-well microtitre plate, and the absorbance was measured at 550 nm in a TECAN Infinite M200 Microplate Reader (GENios-Tecan, Männedorf, Switzerland). The assay was repeated three times in four replicates.

### 2.6. Transmission Electron Microscopy

For transmission electron microscopy (TEM) imaging, *E. coli* WG5 and *P. aeruginosa* (ATCC 10145) suspensions prepared in sterile saline solution (OD_600_ ≈ 0.6) were incubated with the SOM7 coliphage (10^9^ PFU/mL) for 4 h at 35 ± 1 °C and 230 rpm agitation. After incubation, the samples were collected for morphological characterisation via negative staining [27]. For this purpose, a droplet of the uncentrifuged samples was applied to a 400-mesh copper grid supplied with a formvar/carbon film (Sigma-Aldrich, St. Louis, MO, USA). The grid was stained with a 2% uranyl acetate solution for about 1 min and allowed to air-dry. Then, the grid was observed using a Hitachi H-7000 microscope (100 kV, Hitachi Ltd., Tokyo, Japan) at a voltage of 75 kV. Additionally, the samples (10 mL) were centrifuged at 8000× *g* for 5 min at room temperature. After discarding the supernatant and removing any unadhered bacteriophages, the cells were fixed with a glutaraldehyde (2.5%) solution for 2 h at 4 °C, post-fixed with 1% osmium tetroxide–0.8% potassium ferrocyanide solution for 2 h, and finally dehydrated using increasing concentrations of ethanol. Then, the pellets were embedded in EPON resin (EMS, Hatfield, PA, USA) and polymerised at 60 °C for 48 h. Finally, ultra-thin sections of 70 nm in thickness were obtained with a Reichert-Jung Ultracut E ultramicrotome, stained with 2% uranyl acetate and Reynold’s solution (0.2% sodium citrate and 0.2% lead nitrate) [28], and imaged using Hitachi H-7000 TEM at a voltage of 75 kV. All chemicals were purchased from Sigma Chemical Co. (St. Louis, MO, USA).

### 2.7. Data Analysis

Data were reported as the mean value ± standard deviation (S.D.). The significance of the anti-biofilm and biofilm-degrading effect of the SOM7 coliphage against the tested Gram-negative bacteria was determined by an ANOVA test using the SigmaStat 3.5 program (Systat Software Inc., San Jose, CA, USA) at a confidence level of 95%. Tukey’s honest significant difference (HSD) post hoc test was applied when the differences between the measured values were significantly different (*p* < 0.05).

## 3. Results

### 3.1. Antimicrobial Activity of SOM7 Phage

**Figure 1:**
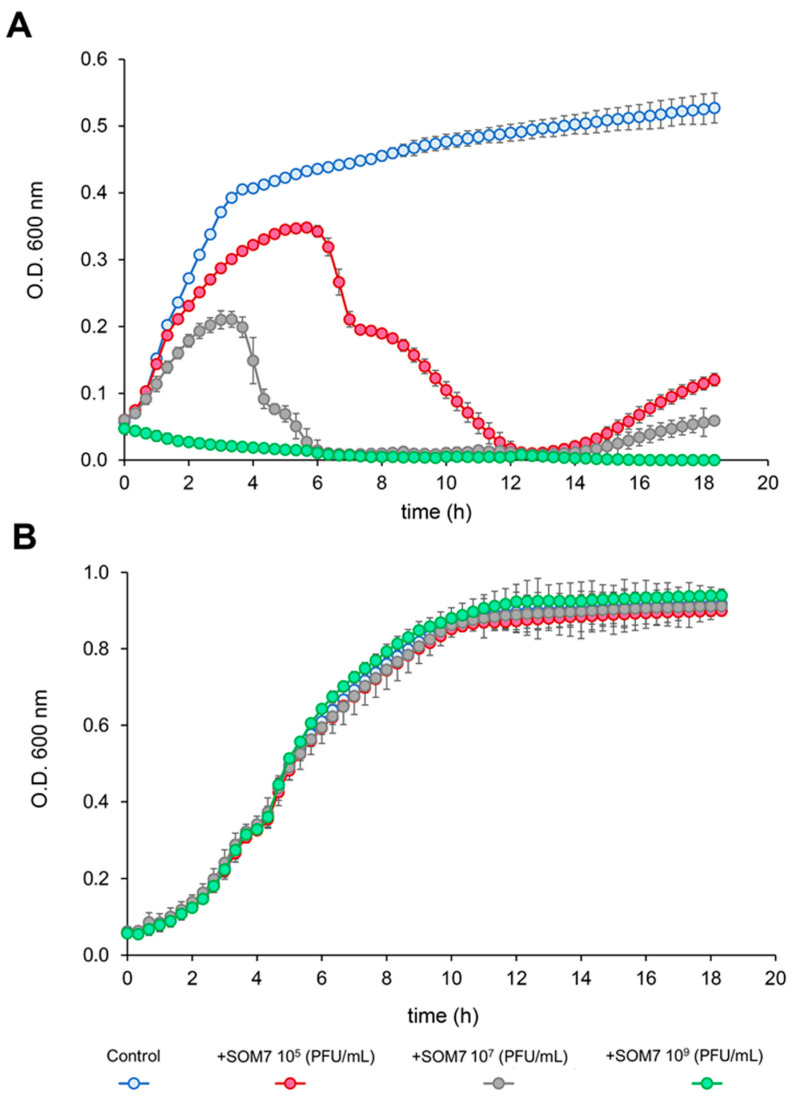
The effect of the somatic coliphage SOM7 on the growth of (**A**) the phage-sensitive *E. coli* WG5 and (**B**) the phage-resistant *P. aeruginosa* (ATTC 10145) strains.

The effect of the SOM7 coliphage on the growth of the sensitive strain *E. coli* WG5 and the non-sensitive bacteria *P. aeruginosa* (ATCC 10145) was tested and confirmed (Figure 1). As expected, the phage-sensitive *E. coli* WG5 was highly affected by the somatic coliphage (Figure 1A) at all phage concentrations used. In addition, no phage-related affect was observed for the SOM7-insensitive *P. aeruginosa* (ATTC 10145) (Figure 1B).

Notoriously, an evident slight growth of *E. coli* WG5 was seen during the first 6 h of cultivation in the presence of 10^5^ and 10^7^ PFU/mL of SOM7 (Figure 1A). This behaviour is related to the “build-up” phase or phage latent period (i.e., the time taken by a phage to reproduce inside an infected host cell) characteristic of a bacteriophage’s infection cycle. During this period, significant lysis of the phage-sensitive bacteria cannot occur until the input phages have undergone multiple replication cycles to reach a concentration high enough to infect most of the cells in the culture. Then, there was a sharp decrease in the OD_600_ (namely the “crash” phase) (Figure 1A), which indicates the ability of the SOM7 coliphage to replicate fast enough and infect and lyse a larger proportion of cells. Finally, a second increase in the OD_600_ value was observed, indicative of the lysogenic growth characteristic of temperate bacteriophages’ propagation strategies [29,30]. This lysis–lysogeny behaviour generally occurs in a situation where a small number of bacteriophages attack a large number of susceptible host cells [31]. In addition, at a higher multiplicity of infection (MOI), the lysogeny propensity decreases, as can be seen in Figure 1A when treating the bacterial cells with a phage titre of 10^9^ PFU/mL.

### 3.2. Anti-Biofilm and Biofilm-Degrading Activity of SOM7 Phage against Non-Target Gram-Negative Bacteria

**Figure 2:**
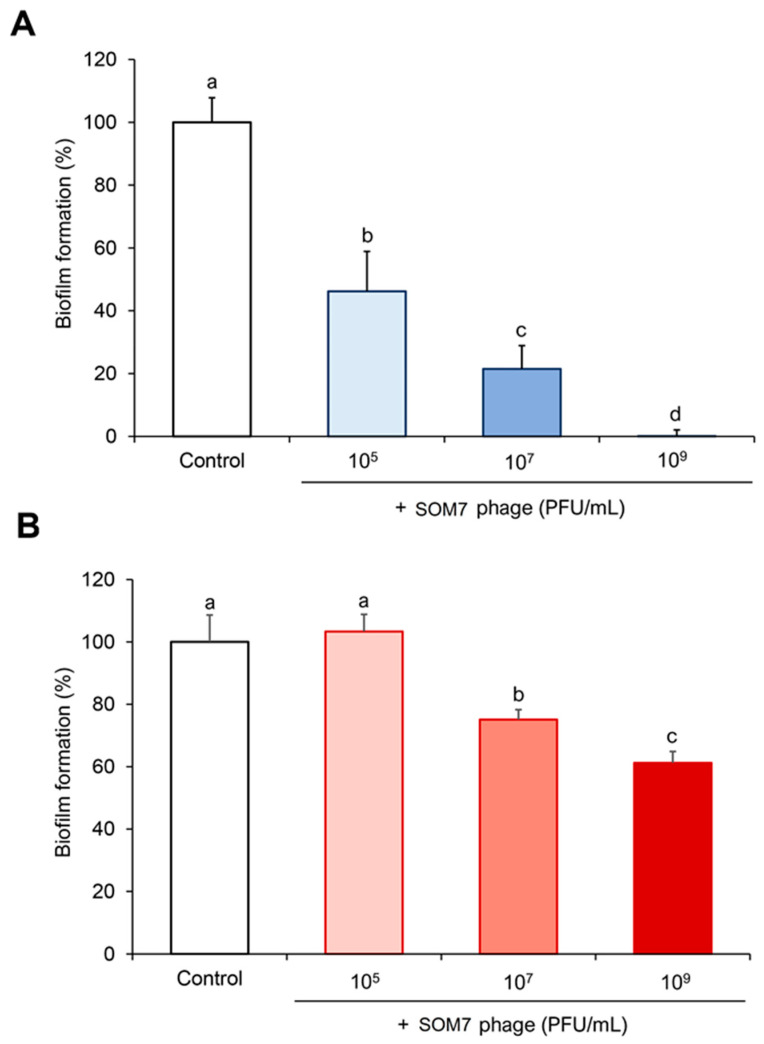
Quantification of the biofilm formed by (**A**) the phage-sensitive *E. coli* WG5 and (**B**) the phage-resistant *P. aeruginosa* (ATTC 10145) growing in the absence (Control) or the presence of different SOM7 concentrations. The bars indicate the standard deviation (S.D.). Different letters represent significant statistical differences (*p* < 0.05) between groups, e.g., “a” and “b” are statistically different from each other.

The most significant alteration in the biofilm-forming ability was observed for the phage-sensitive *E. coli* WG5 (Figure 2A). This strain possesses an attenuated host restriction–modification system and contains only the core part of the lipopolysaccharide (LPS), increasing its susceptibility to coliphages [32]. As can be seen, a significant reduction in biofilm formation (53.8%, *p* < 0.05) was observed for *E. coli* WG5 at a phage titre of 10^5^ PFU/mL, reaching full inhibition of its biofilm-forming capacity when the cells were treated with 10^9^ PFU/mL SOM7 (Figure 2A).

It is noteworthy that SOM7 was also able to significantly (*p* < 0.05) reduce the establishment of biofilms by the non-target *P. aeruginosa* (ATTC 10145) when growing in the presence of 10^7^ and 10^9^ PFU/mL phage. This effect was dependent on phage concentration. At a SOM7 titre of 10^7^ PFU/mL, a 24.9% reduction in the biofilm-forming ability of the non-sensitive *Pseudomonas* was observed, and this value increased up to 38.8% when the cells were treated with 10^9^ PFU/mL phage solution. No biofilm inhibition was observed at the lower phage concentration of 10^5^ PFU/mL (Figure 2B). Thus, these results positively demonstrate the ability of the somatic coliphage SOM7 to significantly reduce the amount of biofilm formed by a non-host Gram-negative strain.

**Figure 3:**
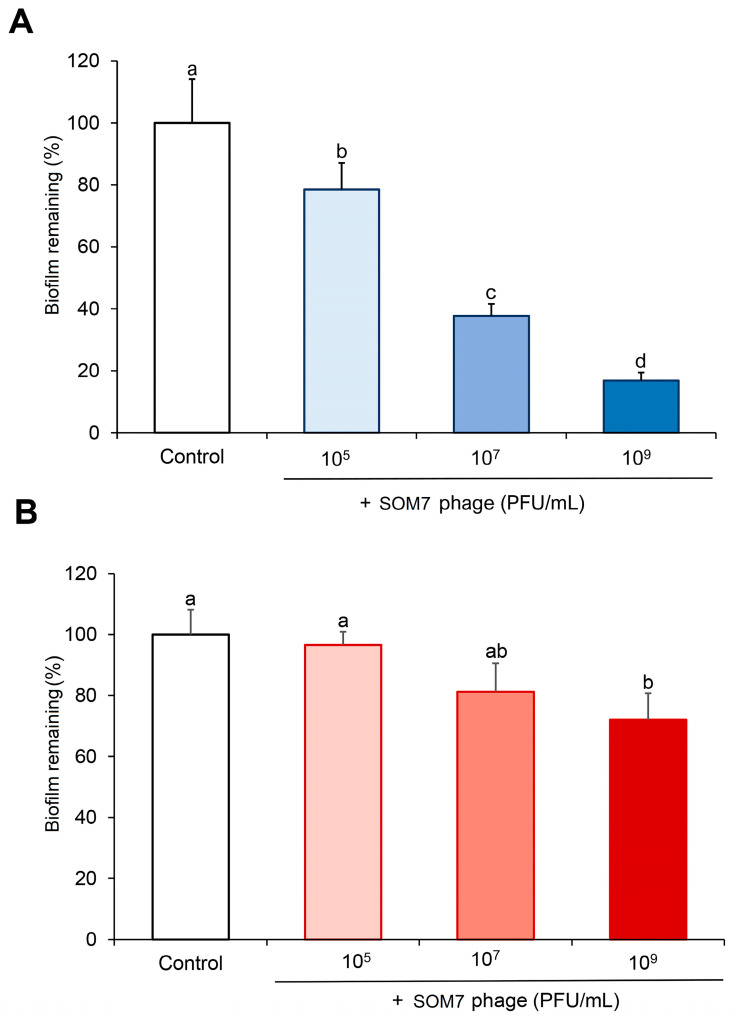
Quantification of the bacterial biofilm remaining after 24 h of treatment in the absence (Control) or the presence of different SOM7 concentrations. (**A**) Phage-sensitive *E. coli* WG5; (**B**) phage-resistant *P. aeruginosa* (ATTC 10145). The bars indicate the standard deviation (S.D.). Different letters represent significant statistical differences (*p* < 0.05) between groups, e.g., “a” and “b” are statistically different from each other but not from “ab”.

Additionally, we also confirmed the ability of the SOM7 phage to eliminate (degrade) pre-established biofilms of both tested microorganisms (Figure 3). The biofilm-degrading ability showed a similar trend to the anti-biofilm effect shown in Figure 2. The somatic coliphage significantly (*p* < 0.05) removed the attached biofilm of the phage-sensitive *E. coli* WG5 at all SOM7 concentrations tested. For this strain, a remarkable increase from 20% to 80% in the cleaning of the bacterial biomass accumulated was achieved when increasing the phage concentration from 10^5^ PFU/mL to 10^9^ PFU/mL (Figure 3A).

Remarkably, a reduced but still noticeable ability to eliminate the amount of biofilm formed by the non-host bacteria *P. aeruginosa* (ATCC 10145) was appraised at a phage titre of 10^7^ PFU/mL (18.8% reduction, *p* < 0.05). This effect was even higher at 10^9^ PFU/mL SOM7 (28% reduction, *p* < 0.05). On the contrary, no biofilm degradation was observed at the lower coliphage concentration tested (i.e., 10^5^ PFU/mL) (Figure 3B), nor in the coliphage-insensitive Gram-positive bacterium tested as a control (Figure S1).

The lowest biofilm-degrading activity quantitated for *P. aeruginosa* (ATCC 10145) (Figure 3B) agrees with the highly notorious persistence of *P. aeruginosa* in clinical settings, which has been attributed to its remarkable ability to form antimicrobial-resistant biofilms [33]. In addition, compared with the results obtained for the anti-biofilm-forming assay (Figure 2), the data from Figure 3 confirm that bacteria living in biofilms are more resistant to antimicrobial agents [7] since lower reduction values were found for the biofilm-degrading assays in both tested bacteria. Finally, these results demonstrate the biofilm-degrading effect of the SOM7 bacteriophage not only against its natural host strain but also for a non-host Gram-negative bacterium.

### 3.3. TEM Assessment of Phage–Bacteria Interaction

**Figure 4:**
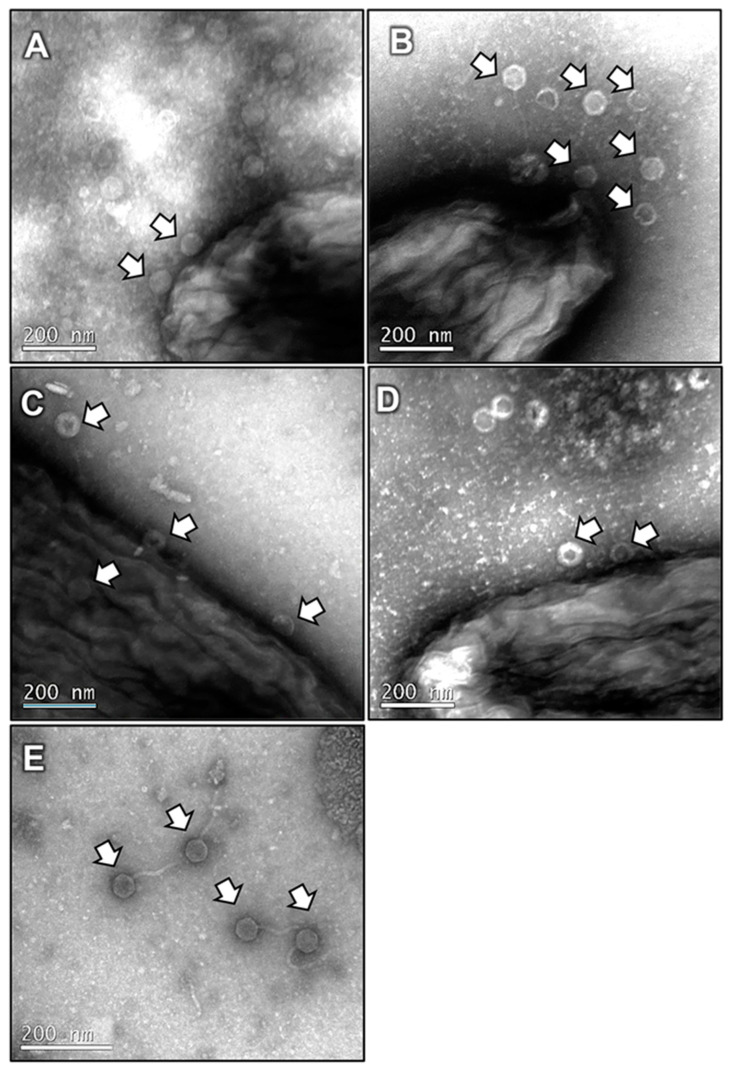
Negative stained TEM images of *E. coli* WG5 (**A**,**B**) and *P. aeruginosa* ATCC 10145 (**C**,**D**) treated with the somatic coliphage SOM7 (**E**). Note the release of phage virions from the sensitive *E. coli* WG5 in (**B**) and the attached phages on the cell surface (white arrows) in (**A**,**C**,**D**). The long, flexible, curly, non-contractile tails and hexagonal heads characteristic of the *Siphovirus*-like phages can be distinguished in (**E**).

To visualise the interaction of the SOM7 phage with bacteria, two different sample preparations for TEM imaging were carried out. For these purposes, a concentrated purified phage solution prepared at the high titre of 10^9^ PFU/mL was used on the premise that there would be an increasing likelihood of phage interaction with the bacterial cells. Negative stained TEM images showed clear evidence of SOM7 attachment to both tested bacteria (Figure 4). A higher proportion of phages could be detected for the phage-sensitive *E. coli* WG5 strain (Figure 4A,B) compared with the number of phages found during the treatment of the non-host *P. aeruginosa* (ATCC 10145) (Figure 4C,D). Besides the expected strong interaction of SOM7 with its target cell, this difference could probably also be due to several infection and replication cycles within *E. coli* WG5, as suggested by the release of phage virions shown in Figure 4B. In addition, the clear phage morphology of SOM7 virions was also visualised (Figure 4E). The cylindrical midsection of the capsid was measured to be approximately 40 nm in diameter. Tail fibres were just observable on unbound phages since the resolution of the bacteriophage structural features decreases upon cell binding; in addition, the bacterial layers cannot be accurately distinguished due to the nature of the negative stain method.

**Figure 5:**
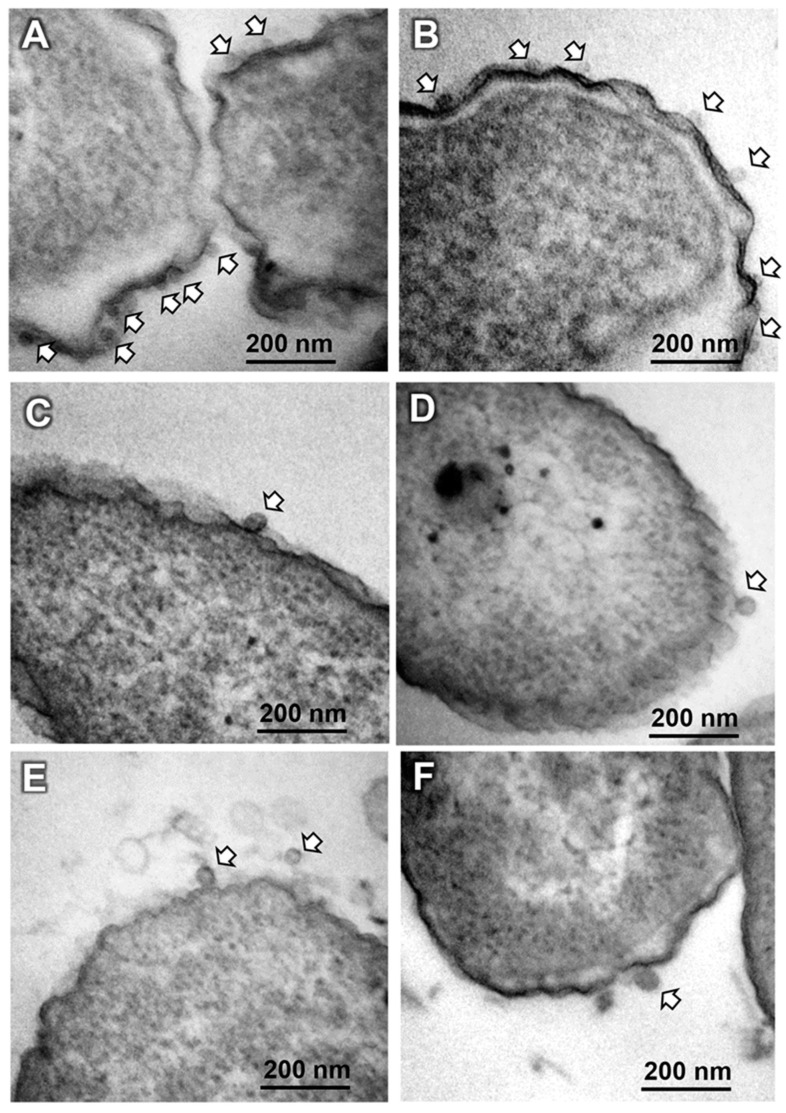
TEM micrographs of ultra-thin sections of *E. coli* WG5 (**A**,**B**) and *P. aeruginosa* (ATCC 10145) (**C**–**F**) treated with SOM7 phage. Virion particles interacting with bacterial cell membranes are indicated by arrows.

On the other hand, the observations from Figure 4 are also supported by TEM images of ultra-thin sections, where SOM7 phages attached to bacteria were visualised as dark geometrical dots (i.e., icosahedral heads) (Figure 5). In these images, the capsid was the sole phage component visible on the surface of the bacterial cells, and the resolution of the phage structural features could not be accurately distinguished due to the limited resolution power of the microscope used. Moreover, considering the multiple washing steps needed for the preparation of the ultra-thin samples, a reduced number of phages remained attached to the cells. However, a higher number of virion particles was observed for the host *E. coli* WG5 compared to the non-sensitive *P. aeruginosa*, probably due to the stronger covalent interaction between phage components and the specific receptors on the cell surface of the sensitive bacteria.

## 4. Discussion

Biofilms are known to be one of the hardest-to-treat bacteria-associated burdens since microorganisms living in communities are more resistant to different antimicrobials. Hence, we tested and compared the ability of a *Siphovirus*-like coliphage (SOM7) belonging to the former *Siphoviridae* family [20,34] to affect the biofilm-forming capacity of a phage-sensitive strain (*E. coli* WG5) and a non-target Gram-negative bacterium (*P. aeruginosa* ATCC 10145). First, we proved the ability of the somatic coliphage to effectively affect the growth of its natural host, *E. coli* WG5 (Figure 1A). In addition, no phage-related affect was observed for the phage-resistant *P. aeruginosa* (ATTC 10145) even at high phage titres (Figure 1B). These results were initially expected due to the intrinsic characteristics of the test microorganisms selected for this study.

Later, the capability of SOM7 to prevent biofilm formation and to degrade the pre-accumulated biomass of its target host was confirmed (Figure 2A and Figure 3A). These results agree with the concomitant SOM7 ability to infect and kill *E. coli* WG5, the reference host strain widely used to detect somatic coliphages [23]. However, our results also proved the exceptional anti-biofilm behaviour revealed by SOM7 against the unsensitive *P. aeruginosa* (ATCC 10145) (Figure 2B and Figure 3B).

The coliphages are a diverse group of phages that infect coliforms and related bacteria and can be classified into different taxonomic groups based on their mechanism of replication, infection mode, morphological characteristics, and genomic content [35]. Siphoviruses are large phages with long, non-contractile tails that infect a wide variety of Gram-negative hosts [36,37]. The infection cycle of most tailed phages begins with sensitive cell recognition through a reversible adsorption process by the first broad-spread “primary” receptor on the potential host (often a sugar motif), followed by irreversible and stronger binding to a “secondary” receptor located on the bacterial cell surface, which subsequently ends in phage DNA injection, triggering the viral replication cycle [35].

Both interactions (i.e., primary and secondary binding) use different and separate structures in the phage tail. The lateral tail fibres of tailed phages first come into contact with primary receptors that are usually bacterial cell surface proteins like OmpC, Tsx, and FadL, but can also be different sugar motifs [38,39,40,41,42]. In the case of Gram-negative bacteria, both lipopolysaccharides (LPSs) and cell wall glycopolymers seem to play similar roles during tailed bacteriophages’ initial adhesion. Surface glycans, such as the highly variable O-antigen or the enterobacterial common antigen (ECA), are the most commonly used primary receptors [11,41]. Therefore, phages can initially attach to different binding sites on the bacterial cell surface, digging for specific receptors that allow them to trigger the infection cycle [13]. If these receptors are found (which occurs just in the case of host bacteria), later, a second more robust and irreversible adsorption process by phage-sensitive bacteria terminal receptors is the one which generates the contraction of the tail sheath [30,34,36,38,40] and, as a consequence, the penetration by the phage tail of the cell envelope and the concomitant viral genome injection via a syringe-like mechanism [35,39,40]. Secondary receptors on Gram-negative hosts are almost exclusively outer membrane proteins of the porin family and/or core LPS sugar structures [38,39,40,41,42,43]. However, not all host receptors are known for newly discovered phages; thus, the exact mechanisms of host recognition by novel phage isolates with a contractile tail are still unclear.

Additionally, the considerable repertoire of glycan-hydrolysing protein domains in the tail fibres of *Siphovirus*-like phages is likely responsible for their exceptionally broad host range and ability to attach to various *Enterobacteriaceae* and Gram-negative strains [39,44,45,46]. For example, it has been suggested that the terminal receptor of the siphophage LL5 seems to be a heptose-linked glucose of the LPS outer core, which is shared by all *E. coli* [44,45]. This observation agrees with the idea that *Siphovirus*-like phages use widespread receptors among Gram-negative bacteria. Therefore, our results from Figure 4 and Figure 5 suggest that non-specific binding of the SOM7 phage to the non-target *P. aeruginosa* can interfere with the establishment of biofilm structures shown in Figure 2. The mechanisms for this interaction involve the adsorption of the SOM7 phage at the lipid bilayer of the bacterial cell membrane, probably through premature bonding to primary unspecific receptors. In agreement with this, it has been shown that tailed phages prepared at high phage titres result in higher phage interaction with liposomes due to attractive Van der Waals forces [47]. Tailed phages were observed to frequently interact with the lipid bilayer and bind to the outside wall of fully formed liposomes and lipid bilayer fragments. Therefore, phage–lipid bilayer interactions may generate a steric hindrance affecting the adherence/attachment of bacteria to abiotic surfaces or even to previously attached microbial cells, interfering with the first stage for proper biofilm establishment (i.e., anchoring of the bacteria to the colonising surface) [47,48].

On the other hand, different synthetic paths to attach bacteriophages to inert polymeric surfaces have been tested. Bacteriophages can be physically adsorbed on Nylon™ polymers, coated surfaces, gold, glass, and plastic materials [49,50,51]. Thus, the adsorption of the SOM7 phage (at high phage titres) onto polymer surfaces could be another possible mechanism involved in avoiding biofilm establishment of the phage-insensitive *P. aeruginosa*. However, phage interactions with synthetic materials are inherently non-favourable unless the attachment is chemically driven [4,5]. In the case of bacteriophages, a suitable covalent attachment to polyethylene (PE) or polytetrafluoroethylene (PTFE) surfaces has been achieved via the carboxylic groups (–COOH) or amine groups present in the phage proteins. In this condition, the authors proved that the bonded phages effectively retained their biological activity against strains of *E. coli* and *Staphylococcus aureus* [5,51]. Therefore, these studies prove that surface attachment of biologically active phages can prevent biofilm formation, thus offering an opportunity for effective eradication of multi-bacterial colonies of pathogens [5].

Quorum sensing (QS) also plays a main regulatory role during biofilm formation in Gram-negative bacteria [11]. This particular phenomenon of microbial communication regulates the metabolic activity of planktonic cells and can induce biofilm establishment. The affect on bacterial adherence caused by SOM7 (Figure 2) could also alter QS signalling [52], retarding biofilm maturation and thus reducing the thickness of the biofilm [48]. In this case, the effect on QS is not directly exerted by the bacteriophage but as a consequence of preventing bacterial biomass accumulation, and this phenomenon is independent of the sensitivity or lack thereof of the target strain. Bacteria can sense the density of themselves or surrounding cells and secrete autoinducers (mainly homoserine lactones in Gram-negative bacteria) into the extracellular space. If the amount of these signalling molecules does not reach a certain threshold, then QS is maintained as “off”, discouraging biofilm formation [48,52].

Another interesting aspect is that most bacteriophages encode different types of cell wall lytic proteins, like depolymerases, endolysins, and virion-associated peptidoglycan hydrolases. These enzymes have the ability to non-specifically degrade the peptidoglycan of all Gram-negative bacteria when they are applied externally. Bacteriophage lytic proteins have been studied for multiple applications (e.g., food safety, pathogen detection/diagnosis, vaccine development), including surface disinfection [53]. Therefore, the potential presence of SOM7-associated hydrolases may be important in destabilising biofilms, even if the phage is not specific for a bacterium and cannot infect it, as suggested by our results from Figure 3.

Biofilms formed by pathogenic microorganisms are well known to be hundreds of times more resistant to antimicrobial agents than planktonic cells, thus making it extremely difficult to treat biofilm-associated infections, even when using high doses of antibiotics, which poses a serious threat to human health [6,7]. Phage-based therapies have the advantage of being safe and non-toxic for human cells, possessing great potential for the removal of pathogenic biofilms [54]. In this sense, phages could penetrate existing biofilms and affect the complex biofilm structure with or without killing the resident bacteria [55]. The mechanisms of biofilm removal using phages are driven by phage-derived lysins and depolymerases [56]. In addition, phages could be structurally engineered or bind with other antimicrobial compounds in combined therapies to enhance microbial biofilm eradication. For example, to evaluate the synergism between phage treatment and chemical disinfection, ref. [57] proved that combining sodium hypochlorite and benzalkonium chloride with phages significantly improved the removal of *P. aeruginosa* biofilms on plastic surfaces. In addition, recent studies have highlighted that combining phages with antibiotics could have superior therapeutic efficacy in increasing bacterial mortality. For instance, ref. [58] demonstrated that the combination of phages with ciprofloxacin exhibited a synergistic effect in eradicating *P. aeruginosa* biofilms in infective endocarditis. Moreover, the effect of treating biofilm infections can also be significantly enhanced when the biofilm is exposed to phages before antibiotic treatment [59].

It has also been proposed that phages can cause a significant reduction in biofilm viability when used in combination with natural antimicrobial agents compared to each treatment alone [60,61,62]. In a recent study, ref. [63] tested the antimicrobial activity of selected essential oils (allyl isothiocyanate, carvacrol, eugenol, and thymol) in combination with *Salmonella* phage-encoding endolysin to control *Salmonella* spread in foods, providing new evidence about the possibility of using a combination of essential oils and phages as synergistic antimicrobials.

In the last few decades, with the development of nanotechnology, nanoparticles have also been described as suitable, efficient, and safer alternatives to antibiotics for pathogenic biofilm eradication [64,65,66,67]. For instance, using a combined strategy, ref. [68] proved that TiO_2_ nanoparticles could promote phage gM13 attachment on the cell surface of *E. coli* TG1, contributing to the entry of the phage.

On other hand, an increasing demand for phage cocktails has recently emerged since a single phage strain often leads to limitations in identifying the fitting strain [69,70]. Phage cocktails could also delay the emergence of phage-resistant bacteria by including multiple phages for bacteria to interact with. In addition, different strains of phages could complement one another by providing the necessary antimicrobial elements that one may be short of [69].

More notably, a recent study demonstrated that phage predation of host bacteria can also have an inhibitory effect on non-phage-targeted species during co-culture [71]. Some other studies have reported phage-mediated effects on non-target bacteria linked to interbacterial interactions and evolved phage tropism for non-cognate bacteria [72,73,74]. So, it is clear so far that our understanding of how phages interact with bacteria, both as individual cells and living in communities (e.g., microbial biofilms), as well as how bacteria respond to phage infection, is still very limited.

Therefore, the results presented here are significant not only for providing fundamental information for the development of therapeutics against mixed biofilms but also for facilitating a better comprehension of phage interactions within host and non-host bacteria. The unique attributes of bacteriophages against non-host bacteria in a situation of excessive concentration levels (high titres) will remain unclear until new evidence appears. Even recognising that each case should be analysed individually, this pioneering work shows for the first time the anti-biofilm- and biofilm-degrading ability of a somatic coliphage against a non-target bacterium. While further studies are needed in other biofilm models and at different phage/bacteria ratios, our results suggest that bacteriophages can be an alternative to achieve the desired anti-biofilm activity in non-susceptible strains, with potential applications in the medical and environmental fields.

## 5. Conclusions

Bacteriophages hold great potential for impeding the biofilm-forming activity of pathogenic bacteria due to their unparalleled diversity. Most of this knowledge usually refers to phage applications against host bacteria since host specificity is a distinctive feature for phage infection. Additionally, fundamental knowledge on the molecular mechanisms underlying phage–host interactions and infection is confined to a few traditional model systems. Our results here prove the ability of a somatic coliphage (siphovirus) to partially prevent and even degrade the biofilm formed by a non-target *Pseudomonas* strain. We suggest that this behaviour could be related to preliminary non-covalent interactions between proteins or different moieties at the page tail and widespread molecules at the bacterial cell membranes. Moreover, we confirmed by electron microscopy this interaction of the coliphage with the surface of the non-host strain in a situation of high phage load. Therefore, the presented results are important for offering new insights into the therapeutic potential of bacteriophages against mixed biofilms and for introducing novel evidence regarding non-lytic interactions between phages and non-sensitive strains. Certainly, the true potential encoded by these bacteria-infecting viruses remains untapped, and bacteriophages for therapy and other biotechnological applications still need to be discovered.

## Data Availability

The authors confirm that the data supporting the findings of this study are available within the article.

## Supplementary Materials

The following supporting information can be downloaded at: https://www.mdpi.com/article/10.3390/biomedicines12102291/s1, Figure S1: Quantification of the bacterial biofilm remaining after 24 h of treatment in the absence (Control) or the presence of SOM7 (10^9^ PFU/mL) or sodium dodecyl sulphate (SDS, 0.5% *v*/*v*) for the Gram-negative bacteria *E. coli* WG5 (phage-targeted bacterium) and *P. aeruginosa* ATTC 10145 (non-target bacterium) and the Gram-positive *S. aureus* ATCC 25923 (coliphage-insensitive bacterium). The bars indicate the standard deviation (S.D.) Different letters represent significant statistically differences (*p* < 0.05) against the control group for each bacterium. Note the positive effect of the SDS detergent (a non-phage treatment) in fully removing the bacterial biofilm in all tested strains.
