# Supplementary material for: Biofilm Prevention and Removal in Non-Target Pseudomonas Strain by Siphovirus-like Coliphage
Source: Biomedicines. 2024 Oct 9;12(10):2291. doi: 10.3390/biomedicines12102291 (PMC11504082; doi:10.3390/biomedicines12102291)
Supplement: Supplementary file 1 [file biomedicines-12-02291-s001.zip › biomedicines-3251269-supplementary.pdf]

Supplementary Material

Biofilm Prevention and Removal in Non-Target *Pseudomonas* Strain by *Siphovirus*-like Coliphage

Leonardo Martín Pérez, Olesia Havryliuk, Nury Infante, Maite Muniesa, Jordi Morató, Ruslan Mariychuk and Tzanko Tzanov

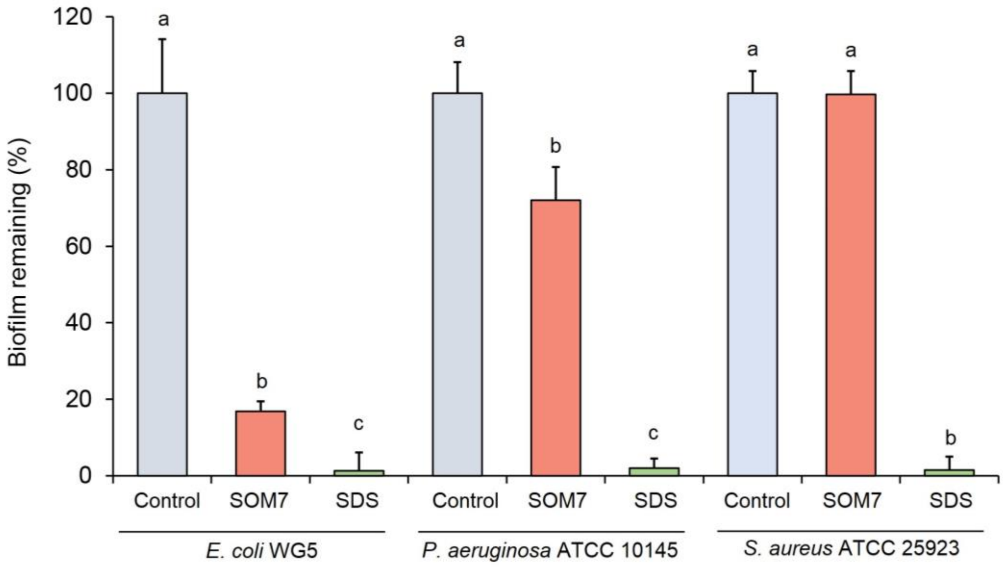

Figure S1. Quantification of the bacterial biofilm remaining after 24-h treatment in the absence (Control) or the presence of SOM7 (10^9 PFU/mL) or sodium dodecyl sulphate (SDS, 0.5% v/v) for the Gram-negative bacteria *E. coli* WG5 (phage-target bacterium) and *P. aeruginosa* ATTC 10145 (phage non-target bacterium), and the Gram-positive *S. aureus* ATCC 25923 (coliphages insensitive bacterium). Bars indicate standard deviations (S.D.). Different letters represent significant statistically differences ($p < 0.05$) against the control group for each bacterium. Note the positive effect of the SDS detergent (a non-phage treatment) in fully removing bacterial biofilm in all tested strains.
